# Design and testing of an optical instrument for skin flap monitoring

**DOI:** 10.1038/s41598-023-44017-6

**Published:** 2023-10-05

**Authors:** Aldo Moreno-Oyervides, Luis Díaz-Ojeda, Oscar E. Bonilla-Manrique, Jorge Bonastre-Juliá, Carlota Largo-Aramburu, Pablo Acedo, Pedro Martín-Mateos

**Affiliations:** 1 Department of Electronics Technology, Universidad Carlos III de Madrid, 28911 Leganes, Madrid Spain; 2 Departamento de Cirugía Plástica, Reparadora y Quemados, Hospital Universitario La Paz, 28046 Madrid, Spain; 3 Departamento de Cirugía Cardiovascular, Hospital Universitario Puerta de Hierro, 28222 Madrid, Spain; 4 Experimental Surgery Service, Hospital Universitario La Paz, 28046 Madrid, Spain

**Keywords:** Optical sensors, Biomedical engineering

## Abstract

Flap procedures are complex surgical tools widely used in reconstructive surgery. Flap ischemia is one of the most dangerous complications, both during the surgical procedure and during the patient's recovery, which can quickly lead to tissue necrosis (flap loss) with serious medical and psychological consequences. Today, bedside clinical assessment remains the gold standard for flap monitoring, but timely detection of flap ischemia is a difficult and challenging task, so auxiliary techniques are needed to support flap monitoring. Here we present a prototype of a new optical diagnostic tool, based on visible light absorption in diffuse reflectance spectroscopy, for non-invasive, continuous, real-time monitoring of flaps. The proposed approach is assessed by monitoring flap ischemic scenarios induced on pig animal models. The results obtained support that the proposed approach has great potential, not only for prompt detection of ischemia (in seconds), but also for clear differentiation between an arterial occlusion and venous occlusion.

## Introduction

The flap procedure is a routine option in reconstructive surgery that is widely used to restore the integrity of the human body after trauma, health complications such as tumor extirpation and infection, or some congenital or acquired deformity^1–5^. Basically, the procedure involves transferring tissue from a donor site, healthy tissue, to the area of a defect, recipient site. A flap is removed with its own vessels (vascularized tissue) and can either be transferred while still attached to its original blood supply (pedicled flap) or physically detached from its original blood supply and reconnected at the recipient site through microvascular surgery (free flap), so that the transplanted tissue always has an adequate vascular supply to receive the necessary molecules to metabolism. The continued supply of oxygen and blood to the flap during the postoperative period is critical for a successful recovery, as a lack of these molecules can quickly lead to tissue necrosis, which can have serious adverse medical and psychological effects on the patient. Vascular failure is the most common and serious complication of the flap procedure (flap ischemia), with irreversible effects in a short period of time, and may occur due to external compression, kinking, or obstruction of the major blood vessels^6,7^. In this regard, early detection of flap ischemia significantly increases the chances of successful recovery, especially when salvage surgery is required, and an appropriate monitoring technique that allows for direct, simple, rapid, accurate, and reliable assessment is critical to this task.

Nowadays, the gold standard for flap monitoring is physical examination (clinical monitoring) by observing tissue color, swelling, and temperature, along with other clinical procedures such as pinprick, skin turgor, and capillary refill^6,8,9^. However, the variability in clinical judgment (relative perception and self-experience), makes it difficult to establish a standardized management protocol, and the need for training to perform appropriate close monitoring (typically monitored 30–60 min during at least 3 days after surgery) are significant limitations in the postoperative care of patients. Even among trained healthcare professionals is sometimes difficult to reliably determine whether the physical examination findings are signs of vascular compromise. For this reason, clinical monitoring is often accompanied by additional medical devices to support postoperative monitoring and achieve more reliably and timely detection of flap ischemia.

There are currently many techniques that have been combined with clinical evaluation for safer and more effective flap monitoring, such as external (handheld) doppler readings (manual) and by means of implantable probes, color duplex ultrasonography, microdialysis, and near-infrared and visible light spectroscopy, among others^9–14^. Handheld doppler readings is the most popular, but presents significant drawbacks, such as low specificity, and usually requires trained healthcare professionals. The other techniques mentioned above also present significant disadvantages such as their excessive cost, lack of simplicity of use and interpretation, difficulty in establishing specific criteria for diagnosis, the requirement of invasive application, and, in some cases, have not completely demonstrated that they really improve the outcome compared to clinical monitoring alone. Therefore, the comparison between the different proposed techniques continues to be a subject of great discussion in the surgical literature, leaving still obvious room for new or improved techniques that alleviate the above-mentioned drawbacks.

In this situation, it could be argued that, nowadays, Near-Infrared Spectroscopy (NIRS) and Visible Light Spectroscopy (VLS) appear as the most promising approaches for flap monitoring^7,9,11^. These techniques provide with a non-invasive, continuous, real-time assessment, they can be used for remote monitoring, and they have demonstrated that allow for prompt detection of vascular compromise, even better than doppler readings and clinical monitoring. In particular, VLS can be noted for offering greater sensitivity to alterations in tissue oxygenation compared to NIRS. However, despite the promising advantages of both NIRS and VLS, there are still important aspects that need to be addressed in order to achieve full acceptance in flap monitoring by the clinical community.

In this article, a new compact and portable version of an optical diagnostic tool for non-invasive flap monitoring is proposed and tested using Large White pigs as animal model. As presented in this paper, in the tests performed, the proposed optical diagnostic tool, based on light absorption (VLS) in Diffuse Reflectance Spectroscopy (DRS)^15–17^, has allowed not only for the prompt detection of ischemia, but also for the clear differentiation between an arterial and venous occlusion.

## Methods and materials

### Design of the portable optical instrument: architecture and operating principle

As mentioned above, the proposed optical monitoring system is based on light absorption in DRS^15–17^. A probe placed on the surface of the flap directs a beam of light onto the tissue. This light-tissue interaction causes reflection, refraction, absorption, and multiple scattering of photons by components of the dermis and epidermis. The latter two phenomena are critical to DRS. The objective is to recover for its subsequent analysis the light that reemerges from the tissue by means of a photodetector placed on the surface of the flap. Specially, the light-tissue interaction within the dermis layer is very important as in it, hemoglobin is the dominant absorber (in the wavelength range employed in this system), providing information on blood volume and oxygenation, while the epidermis contribution is mainly associated to melanin^15,18^. Thus, light from the dermis can be used to assess not only the total concentration of hemoglobin (of vital importance to the objectives of this work) to differentiate venous from arterial occlusion, but also the volume fraction of oxy- and deoxyhemoglobin. As shown later, a very good level of sensitivity is achieved with a simple and low-cost design using only two wavelengths of visible light (660 nm and 450 nm). As it could not be otherwise, these wavelengths have been carefully selected based on data collected from numerous previous experiments on rat models and other in-house laboratory tests^19^. In summary, rat model studies were performed using the same interrogation technique (DRS) but using a white light source and an extended range spectrometer operating from 400 to 700 nm. The spectral range that showed the greatest variation in diffusely reflected intensity for the two target scenarios (venous and arterial occlusions) extended from 450 to 470 nm. This variation was maximized when a wavelength in the vicinity of 660 nm was employed as reference. Therefore, two LED emitters, covering these two ranges of interest, 450 nm and 660 nm, were chosen. It has to be said that this design decision was finally taken after several experimental measurements, using the wavelengths preliminarily selected, and evaluating various parameters, such as consistency and sensitivity to changes in tissue hemoglobin concentration, but also noise level and intensity in the recovered signal.

**Figure 1:**
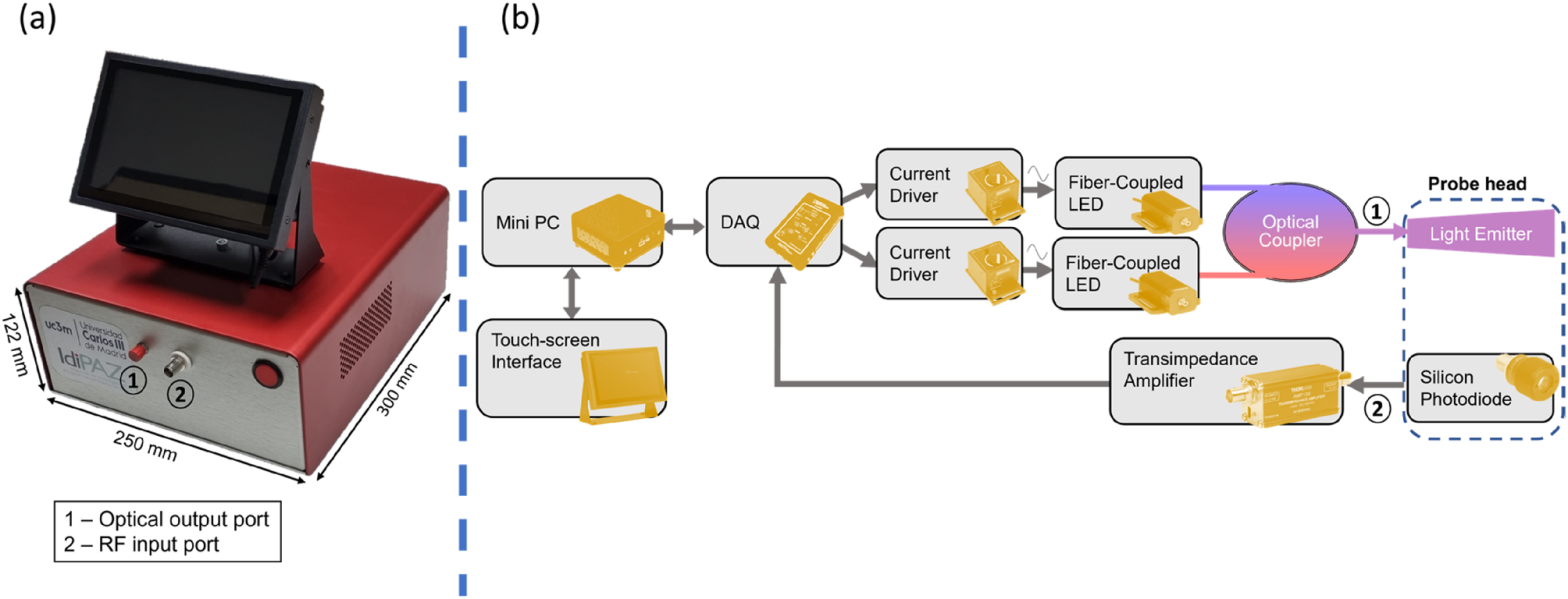
(**a**) Photograph of the optical monitoring system (laboratory prototype). (**b**) Block diagram describing the main components of the optical instrument.

A photograph of the demonstrator of the optical monitoring system is shown in Fig. 1a, together with a block diagram describing its main components shown in Fig. 1b. As shown in the block diagram, the light from two fiber-coupled LEDs, that are intensity modulated at different frequencies by two current drivers, are combined by a fiber coupler and taken to an optical output port to which the probe is connected. After the light-tissue interaction, the detected signal is amplified and digitized in real time using an acquisition card. The optical intensities, associated with the two illumination wavelengths used for spectral interrogation, are recovered by a phase-sensitive detector implemented in software.

**Figure 2:**
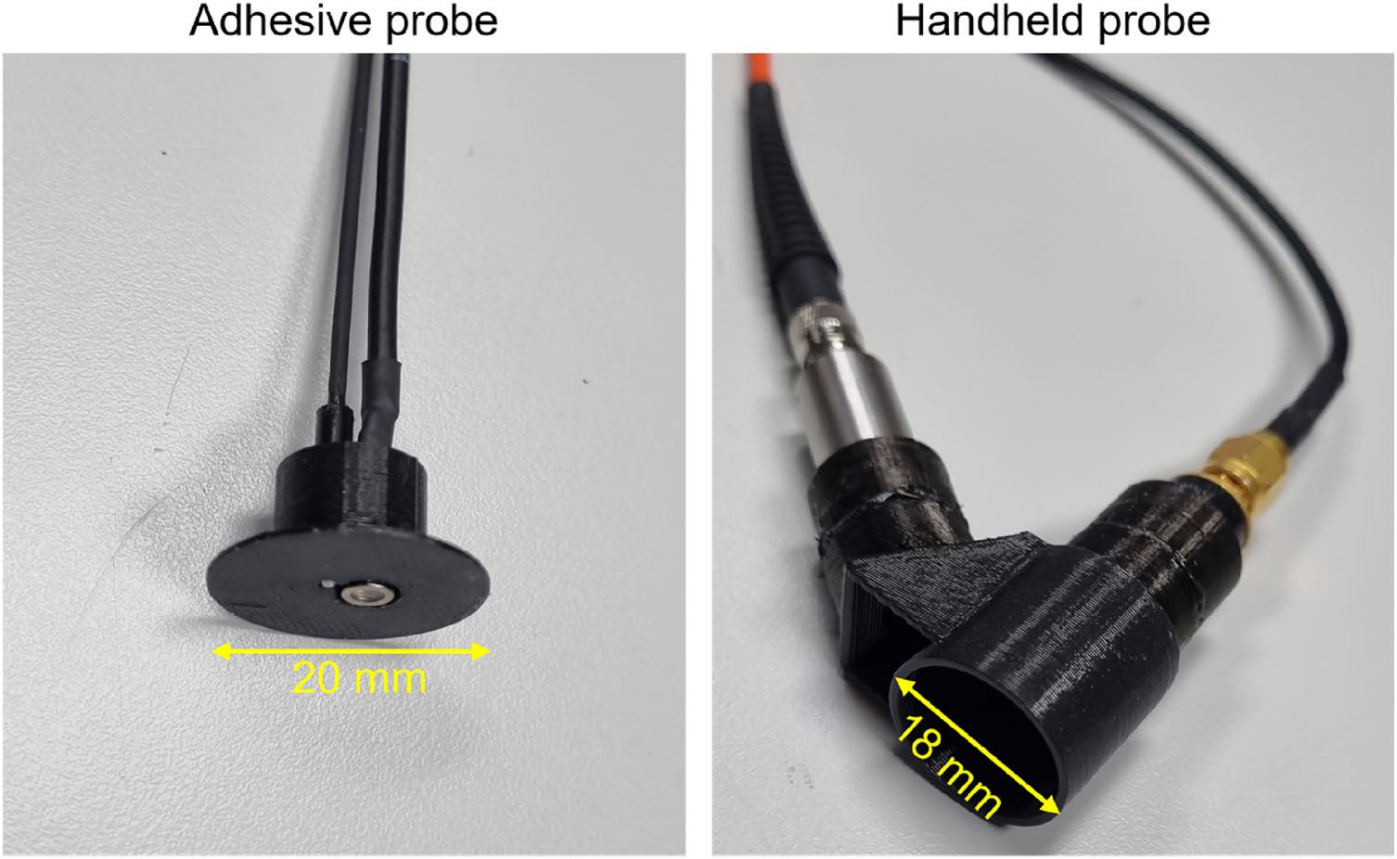
Photographs showing the two 3D designed probe heads used for experimental test.

Two probe heads, shown in Fig. 2, have been designed to evaluate two different monitoring modes. On the one hand, a probe with a head that can be adhered to the tissue surface is shown in the left panel, in which both the sensor (a silicon photodiode) and the illumination (end-ferrule fiber cable) are in direct contact with the tissue. On the second, a probe for handheld readings shown in the right panel has also been developed, that is more appropriate when continuous monitoring is not necessary. In the case of the handheld probe, light from the fiber optic cable is collimated and directed toward the flap tissue surface, illuminating an area of approximately 30 mm^2^. Then, specular reflected light from the tissue surface and single scattering light from the epidermis layer is removed in order to maximize the contribution from the diffusely reflected light in dermis layer. To that aim, we use two crossed polarizers that are placed between the collimator and the sensor^20–23^ and Brewster’s angle illumination (considering the skin refractive index^23^) to minimize the contribution of those unwanted photons.

Finally, the recovered intensities from each wavelength (660 nm and 450 nm) are processed and used for flap condition monitoring. The acquisition card, employed for frequency modulation of the LEDs and signal digitalization, is controlled by a software running in a minicomputer integrated into the system enclosure, providing a user-friendly interface for system operation and data visualization through a touch screen (Fig. 1).

### Experimental test: sample and protocol

In order to evaluate the optical monitoring system in a highly relevant scenario, as close as possible to clinical practice, an animal model of pedicled flap based on direct cutaneous perforator in pig was prepared for system evaluation. Specifically Large White pigs were employed. Pig skin is widely recognized as the most similar to human skin due to its similar structure to human epidermal thickness and dermal-epidermal thickness ratio and similar physical and molecular responses to various growth factors, among other characteristics, that have made it the standard model for several skin studies^24^. The experimental measurements were performed at the Experimental Surgery Unit of Hospital La Paz Institute for Health Research (IdiPAZ), which provided the handling, housing, and surgical facilities for the animal model.

The pig was sedated in its cage in the animal facility with an intramuscular injection of Ketamine (12 mg/kg) mixed with Midazolam (0.5 mg/kg) for safe transfer to the operating room. Anesthesia was induced by inhalation of sevoflurane (8%), and during surgery and measurements he was continuously maintained with intravenous Propofol (11 mg/kg/h) mixed with Fentanyl (3 µg/kg/h). The pig's temperature, pulse oximetry, capnography, and blood pressure were monitored continuously, the latter being controlled by doses of norepinephrine. Experimental tests were carried out on four pedicled flaps performed on two animals, the trials were conducted over a period of two days and a several measurements were taken. A pedicled flap was used for testing because it allows for a more efficient use of operating room time while providing results directly comparable to a free flap scenario.

**Figure 3:**
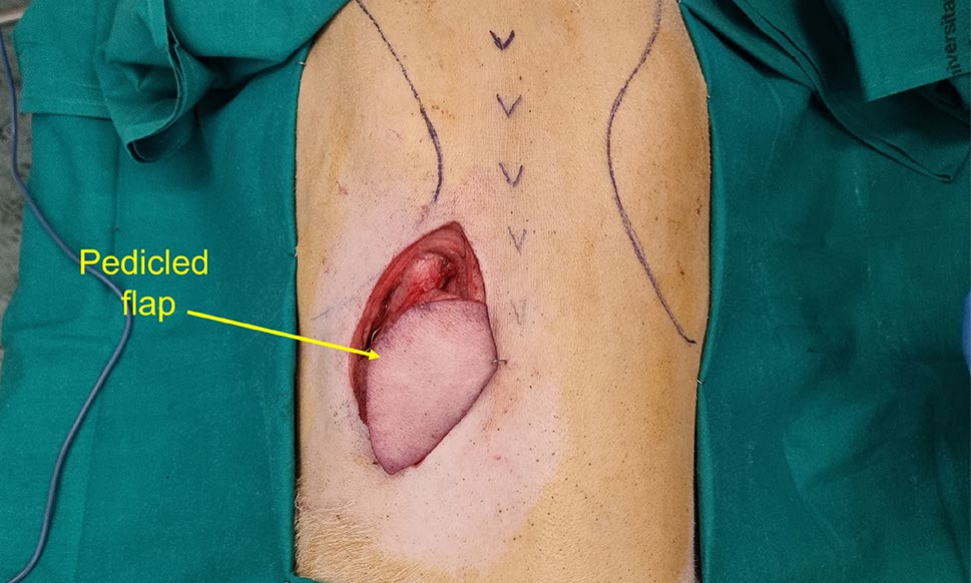
Photograph of the animal model taken in the operating room before the measurements.

As shown in Fig. 3, a pedicled flap based on direct cutaneous perforator was designed in order to provide a test scenario for the monitoring optical system. The tissue was entirely removed from the body, leaving a single vascular pedicle (artery and vein). Flap ischemia was induced by applying an occlusive microvascular clamp to either the artery or vein of the flap's vascular pedicle in order to evaluate the performance of the optical monitoring system. Measurements were repeated for both monitoring system probe heads.

### Ethics declaration

All experimental procedures were carried out in accordance with the ARRIVE guidelines and with the current regulation RD 53/2013 about the protection and use of animals in scientific research, and approved by the Ethics Animal Welfare Ethics Committee of the Hospital La Paz Institute for Health Research (IdiPAZ) according to all external and internal bio-safety and bio-ethics guidelines, and by the Spanish competent authority with registered number PROEX.216.3/20.

## Results and discussion

**Figure 4:**
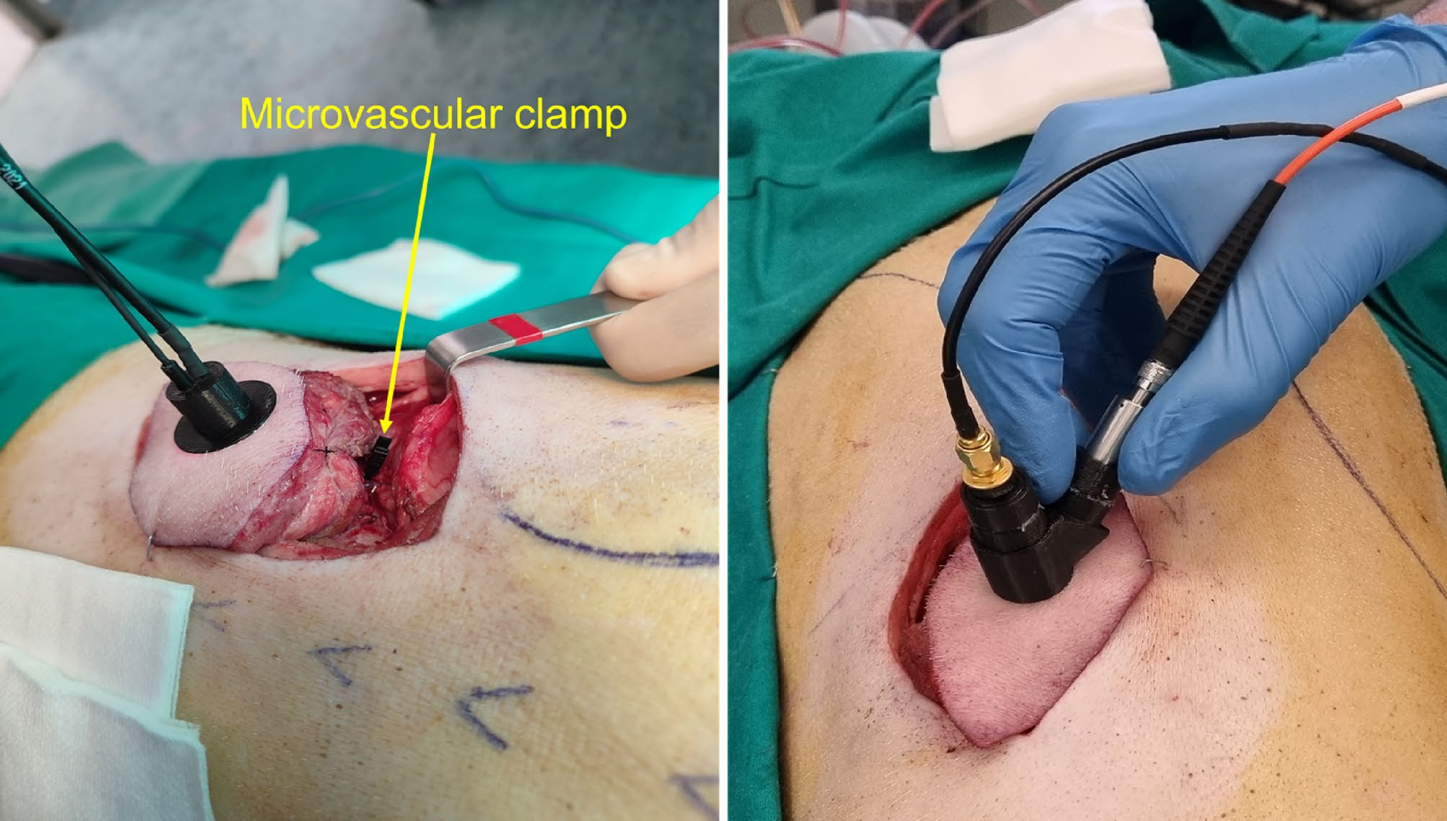
Photograph of the animal model taken in the operating room before the measurements.

As discussed above, the objective of this work was the evaluation of the ability of the proposed optical monitoring system to identify, in real time, arterial or venous ischemia in flaps. For this purpose, ischemic events were induced in vein and artery by means of an occlusive microvascular clamp while the flap was continuously interrogated with the optical monitoring system. As illustrated in Fig. 4, the response of the system to several ischemic events was measured using the two probe heads described above. Prior to applying the microvascular clamp, a calibration was performed to normalize the basal state of the flap tissue. This implies that the intensity of the optical signal re-emerging from the tissue at the two measuring wavelengths is recorded and the ratio of intensities at the two signals (660 nm and 450 nm) is used as the baseline level. Once this has been done, changes in the value of the ratio with respect to this reference point are monitored and used as an indicator of the ischemic state of the flap.

**Figure 5:**
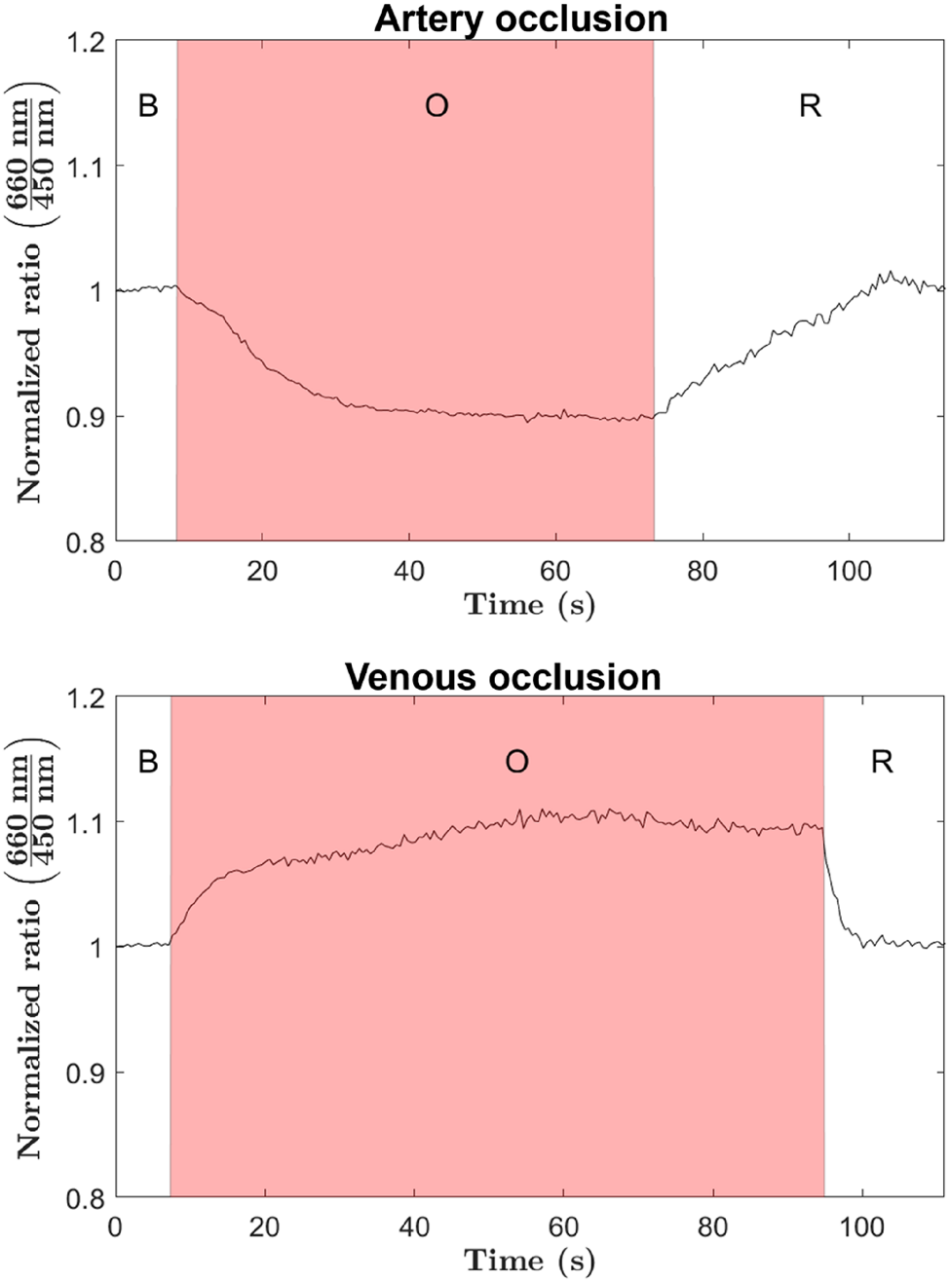
Registered trajectories of the estimated indicator by using the hand-held probe during both ischemic scenarios. For each measurement three different periods are identified: baseline period (B), occlusion period (O), and recovery period (R).

Figure 5 shows two curves corresponding to the evolution of the estimated indicator over time during artery and vein ischemia using the handheld probe. Three periods can be identified in all measurements, corresponding to the following order: the basal situation of the flap (B), the occluded period (O), shaded in red, and a recovery period (R) when the microvascular clamp is removed. As it can be seen, once the microvascular clamp is applied, the optical monitoring system quickly reacts to the sudden change in hemoglobin concentration. The indicator shows strong opposite trends in the case of venous or arterial occlusion, providing a clear indication of increased or reduced tissue hemoglobin concentration, respectively. Although the occlusion is complete, the trajectory of the indicator takes a few seconds to reach an asymptotic trend. This is due to the process of evacuation of blood through the venous system after occlusion of the artery, or due to the accumulation of hemoglobin in blood vessels in the case of venous occlusion (passive hyperemia). Then, once the microvascular clamp is removed, the indicator clearly shows how the basal condition of the flap tissue is reached due to the recovery of the blood perfusion. It can also be observed that arterial occlusion has a longer recovery time than venous occlusion. This is probably due to an internal dilatation of the blood vessels contained in the flap tissue during the period of ischemia, which is known as reactive hyperemia^25,26^. To the authors’ view, these results provide an utterly clear illustration of the capabilities of the tool to detect, not only ischemic events, but also to identify whether the occlusion occurs in the vein or artery.

**Figure 6:**
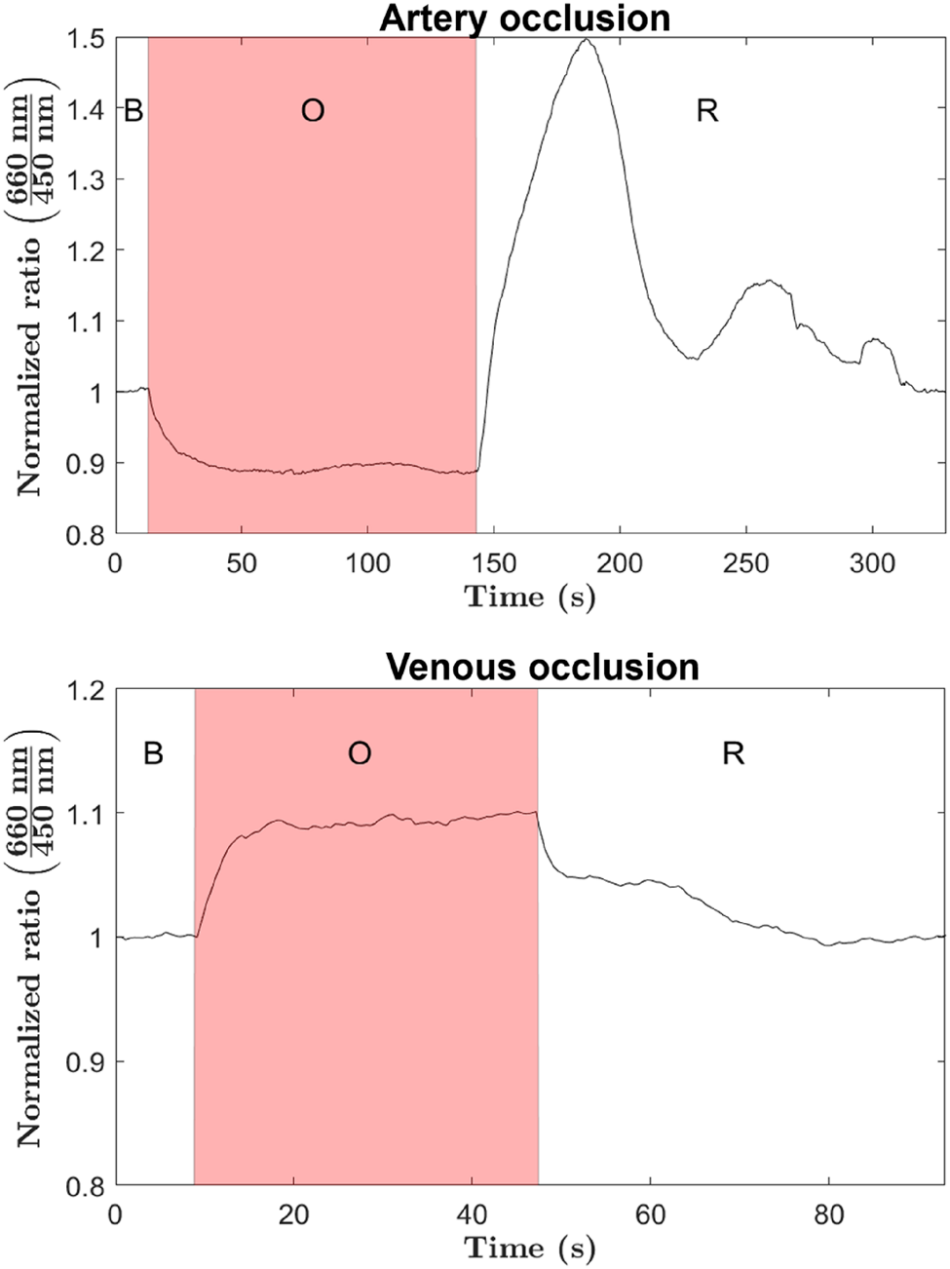
Registered trajectories of the estimated indicator by using the adhesive probe during both ischemic scenarios. For each measurement three different periods are identified: baseline period (B), occlusion period (O), and recovery period (R).

Both ischemic scenarios shown in Fig. 5, were re-evaluated using the head probe designed for continuous measurement, previously referred to as the adhesive probe. The two registered curves corresponding respectively to the venous and arterial ischemic events are shown in Fig. 6. As it can be seen, the trajectories of the indicator after application of the microvascular clamp are very similar to those shown in Fig. 5, further supporting the ability of the system to clearly detect venous and arterial ischemic events. In addition, the adhesive probe, with the light emitter and detector in full direct contact with the tissue, allows to see in greater detail the reactive hyperemia that occurs after the microvascular clamp is removed from the artery. Reactive hyperemia consists of a transient increase in blood flow (temporarily reaching a level above basal flow) that occurs immediately after release of an occluded artery, with the magnitude and duration of the transient directly related to the duration of ischemia. Such hyperemic response can be clearly followed in Fig. 6 (top panel), showing an increase in tissue hemoglobin concentration above basal level after release of the occluded artery (peak value of 1.5), followed by two smaller transients, presumably related to an autoregulation process (myogenic mechanism), until the basal level is reached. More extensive studies, including biochemical analysis, could explain in detail the smaller transients observed in the measurement. It should be clarified that the two probes developed have, in practice, very different penetration depths, and this is the reason for the clearly differentiated response to ischemic events.

**Figure 7:**
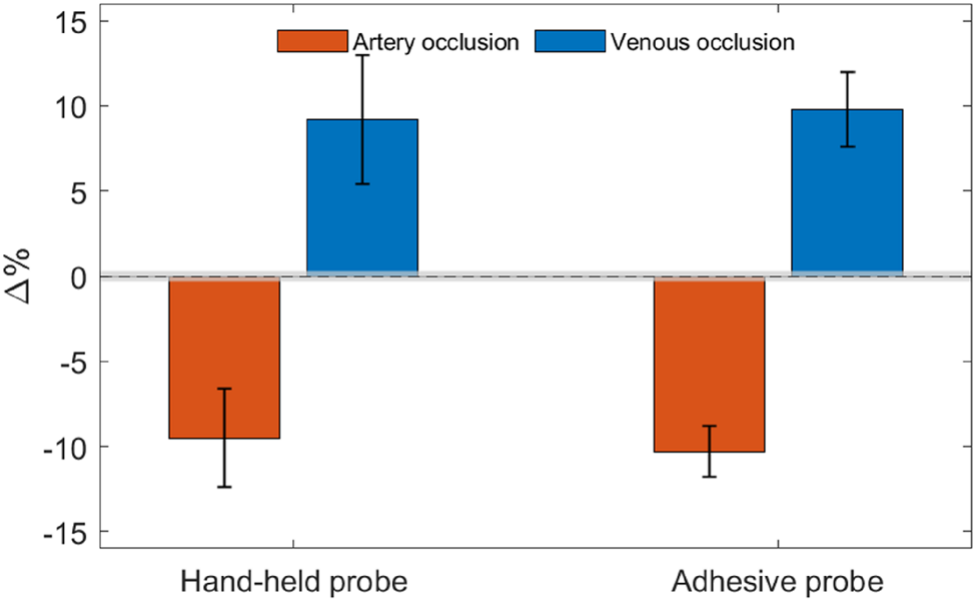
Bar plot illustrating the percent change of the indicator from baseline for each scenario and head probe. The gray shaded area shows the standard deviation of the noise at the baseline. Data corresponding to this figure are shown in Table 1.

A total of 28 measurements were recorded using both the handheld probe and the adhesive probe for arterial and venous occlusion. The results obtained are summarized in Table 1, showing the mean change in the indicator from baseline for each scenario with the corresponding standard deviation and baseline noise. This information is better illustrated by the bar graphs in Fig. 7. As can be seen, the mean percentage change from baseline is consistent and well above the baseline noise, demonstrating excellent performance in detecting vein or artery occlusion. It should be noted that in none of the measurements performed was there a case of a failure to identify an occlusion scenario, i.e., the change in indicator from baseline was always negative for arterial occlusion and positive for venous occlusion.

**Table 1:** Statistical summary of measurements performed on four equally prepared flaps in the two pigs.

Probe head	Scenario	Sample size (n)	Baseline noise (std σ)	Indicator variation (mean µ ± std σ)
Hand-held	Arterial occlusion	8	0.2%	− 9.5 ± 2.9%
	Venous occlusion	7	0.19%	9.2 ± 3.8%
Adhesive	Arterial occlusion	6	0.21%	− 10.3 ± 1.5%
	Venous occlusion	7	0.18%	9.8 ± 2.2%

In addition, and with the aim of further exploring the operation range of the tool, both venous and arterial ischemic conditions were reproduced after an increase in the pig's blood pressure was induced by administration of 200 µg of adrenaline. Fluctuations in tissue hemoglobin concentration after venous or arterial occlusion follow the same hemodynamic pattern observed in previous measurement. Regarding the arterial occlusion, the change in the indicator from the baseline was much greater (more than double), and with a more stable asymptotic trend. On the other hand, small cyclic fluctuations within the asymptotic trend can be observed during venous occlusion, which were not registered in the previous measurement. Such small cyclic fluctuations may be due to the rhythmic contractions of the arterioles (vasomotion)^27^.

**Figure 8:**
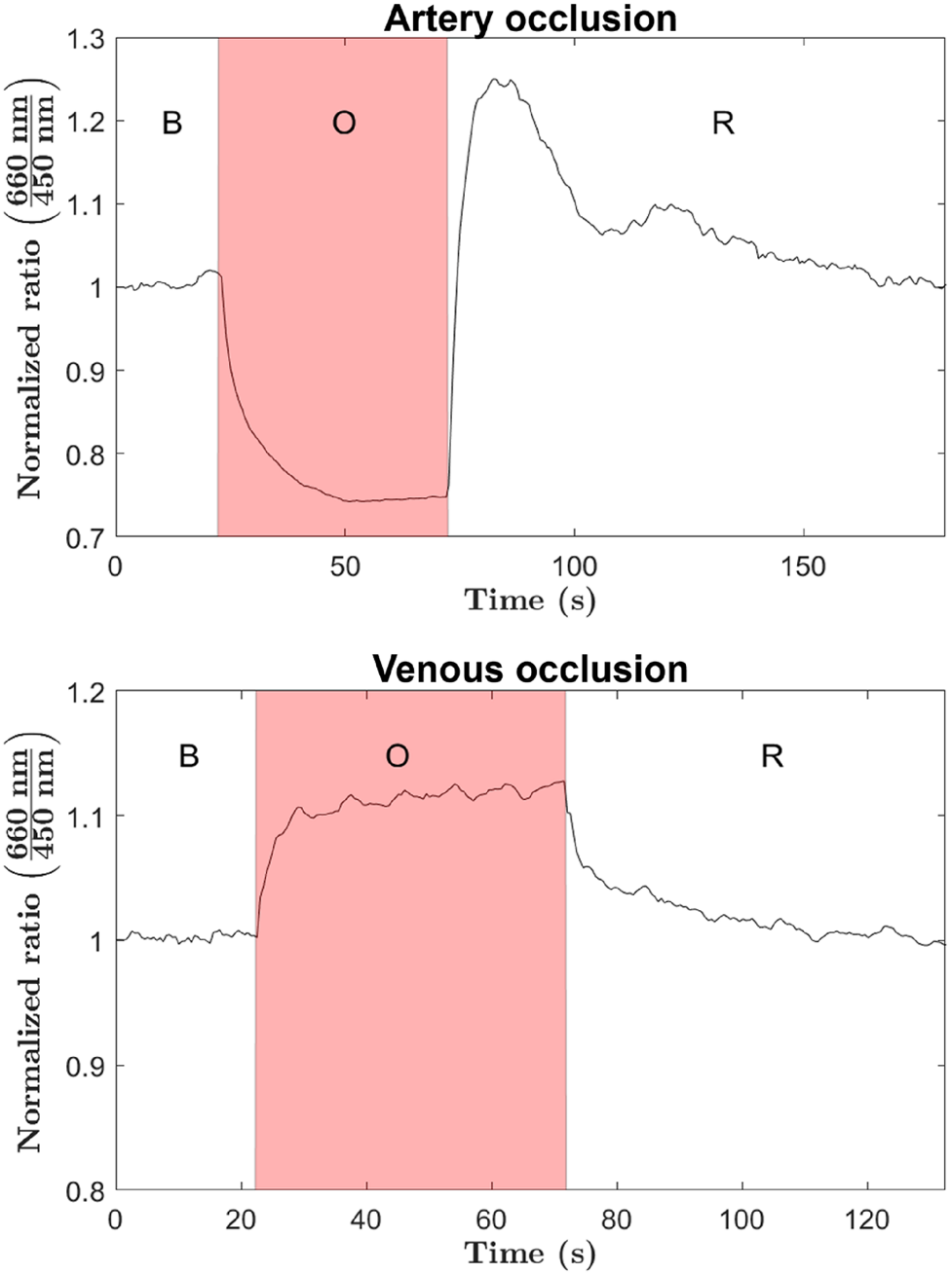
Registered trajectories of the estimated indicator by using the adhesive probe during both ischemic scenarios with increased blood pressure. For each measurement three different periods are identified: baseline period (B), occlusion period (O), and recovery period (R).

Finally, it should be noted that the known dependence between the magnitude and duration of the reactive hyperemia and the duration of the ischemia can be observed by comparing the measurements corresponding to arterial occlusion shown in Figs. 6 and 8. The reactive hyperemia recorded in Fig. 6, involving an ischemia period of approximately 130 s, has greater magnitude and larger duration than the observed in Fig. 8 (50 s induced ischemia). This measurements not only allows to validate the indicator's ability to closely follow changes in tissue hemoglobin concentration and other hemodynamic phenomena (such as the known dependence between the magnitude and duration of reactive hyperemia and the duration of ischemia), but mainly to show how a small increase in blood pressure can significantly enhance the performance of the system, which may, in some cases, be an alternative to improve measurement.

All the captured hemodynamic patterns, associated with the arterial and venous occlusion, by the optical monitoring system are clear evidence of the great potential of the proposed approach, providing a reliable and effective indicator for the detection of arterial ischemia and venous ischemia in flaps, which can be used for handheld readings or continuous monitoring.

The authors believe that a comparison of the proposed system with comparable non-invasive alternatives that are commonly used for perfusion measurement today will provide a better insight into the characteristics and capabilities of the instrument. It should be made clear that the device developed in this work is a technological demonstrator, which in no way maximizes the performance achievable by the proposed method. However, as this section shows, it has provided very respectable results.

To start the discussion with hyperspectral imaging systems, given their current importance, it can be assured that the proposed system has far superior performance characteristics in terms of measurement noise and sensitivity to hemodynamic changes. Firstly, the spectral power density of the LED sources used is (or can easily be) far superior to that of the white light sources commonly used for hyperspectral imaging systems. As very specific spectral characteristics (in terms of spectral bandwidth) are used here for perfusion measurement, the number of photons that can be injected into the tissue within the volume of interest is much higher. Similarly, the power that can be captured by a point detector, especially in the case of large area photodetectors, is orders of magnitude greater than that captured by the pixels of a camera. The same applies to noise, which is much easier to limit on a point detector than on a two-dimensional sensor. With regard to the presented system, these facts are confirmed by the baseline noise data provided in Table 1, where noise levels are notoriously low. Besides this, the availability of an image of the flap, rather than a measurement at a specific point, is not a particular advantage for the objective that the system presented in this article is intended to achieve.

The same factors from the previous discussion apply to both NIRS and VLS; the higher power spectral density of the sources used and the better photon capture capabilities of the point detectors compared to a spectrometer mean that the performance of the proposed system can be much higher (in fact, the studies that preceded the development of the system presented in this article were performed with such systems). Compared to these methods, the main shortcoming of the proposed DRS system is its inability to provide tissue oxygen saturation, which may be useful in some situations (although it is true that this is a measure that can be achieved by increasing the number of interrogation wavelengths of the system). Nonetheless, an important aspect that distinguishes the system here presented is the, comparatively very low, cost of implementing a potential final commercial instrument, as both hyperspectral systems and optical spectrometers are notoriously expensive pieces of equipment. It could be added that an advantage common to all the systems analyzed above is that they do not require specially trained professionals to interpret the results obtained, which are a clear representation of the hemodynamic scenario present in the measurement area.

Another technique that has shown promising results in free flap monitoring is Laser Doppler Flowmetry (LDF), although this method offers a better ability to detect blood flow, it is much more complex and costly to implement. In addition, it requires mandatory contact operation and does not allow for remote operation, which can be very useful. However, the main drawback of LDF in terms of its use in this application is the difficulty in differentiating between venous and arterial occlusion^28^, which is very useful for flap monitoring at all stages of the procedure and that the proposed DRS method straightforwardly provides in a direct and clear manner.

## Conclusion

In this work, the design of a novel optical instrument based on DRS technique for flap condition monitoring is presented and assessed on Large White pigs. The experimental flap model developed in the pig has allowed evaluation of the optical monitoring system in a scenario very similar to that used in routine clinical practice. Real-time monitoring of the flap during artificially induced ischemic events in the artery and vein was used to evaluate the system's ability to detect ischemia. In addition to promptly detecting arterial and venous ischemic events, several hemodynamic phenomena that are typical of ischemia were unambiguously identified in the monitored flap.

Finally, it should be noted that the optical system used for flap condition monitoring presented in a prototype version can be redesigned into an even more compact and cost-effective commercial version, while maintaining very interesting features such as a user-friendly interface, non-invasive application, easy operation, real-time readings, high sensitivity to tissue hemoglobin concentration, and providing an easy-to-interpret medical indicator. All the above-mentioned features are optimal for the development of a medical diagnostic tool with great impact for assisted follow-up during surgery and recovery of flap procedures.

## Data Availability

The data that support the finding of this study are available from the corresponding author upon reasonable request.
